# The Wnt Target Gene L1 in Colon Cancer Invasion and Metastasis

**DOI:** 10.3390/cancers8050048

**Published:** 2016-05-11

**Authors:** Gal Haase, Nancy Gavert, Thomas Brabletz, Avri Ben-Ze’ev

**Affiliations:** 1 Department of Molecular Cell Biology, Weizmann Institute of Science, Rehovot 76100 Israel; gal.haase@weizmann.ac.il (G.H.); nancy.gavert@weizmann.ac.il (N.G.); 2 Experimental Medicine I, Nikolaus-Fiebiger-Center for Molecular Medicine, University of Erlangen-Nuerenberg, Erlangen, 91054, Germany; thomas.brabletz@fau.de

**Keywords:** Wnt target genes, L1, cell adhesion, colon cancer, invasion and metastasis

## Abstract

The Wnt-β-catenin signaling pathway is highly conserved during evolution and determines normal tissue homeostasis. Hyperactivation of Wnt-β-catenin signaling is a characteristic feature of colorectal cancer (CRC) development. β-catenin is a major transducer of the Wnt signal from the cytoplasm into the nucleus where it acts as a co-transcriptional activator of β-catenin-TCF target genes. β-catenin is also required for linking cadherin type cell-cell adhesion receptors to the cytoskeleton, and consequently Wnt-β-catenin signaling is an attractive system for investigating the role of adhesion-mediated signaling in both normal intestinal tissue homeostasis and CRC development. In this review, we summarize our studies on one Wnt-β-catenin target gene, L1, a member of the immunoglobulin-like cell adhesion transmembrane receptor family. We describe the mechanisms of L1-mediated signaling in CRC cells, its exclusive localization in invasive areas of CRC tissue, and its ability to increase cell motility and confer metastasis to the liver. We discuss the activation (by L1) of genes via an ezrin-NF-κB pathway and the induction of genes also found in the intestinal stem cell signature. By studying L1 (adhesion)-mediated signaling, we expect to learn about mechanisms regulating both normal intestinal homeostasis and CRC development.

## 1. Introduction

Cell-cell adhesion is a fundamental biological process required for the formation of multicellular organisms. Cellular and tissue morphogenesis are defined and tightly regulated by the adhesive interactions between neighboring cells [1]. Numerous studies have addressed the molecular mechanisms underlying the signals conveyed inside the cell by transmembrane cell adhesion receptors and their cytoplasmic binding partners to regulate the expression of downstream target genes. Such genes determine, in turn, cellular and tissue morphogenesis by changes in the adhesive interactions of cells. A tight coordination between adhesion-mediated signaling and gene expression is a characteristic feature of normal tissue homeostasis. Changes in this coordination are often detected in various diseased tissues, mostly in the later phases of cancer development that are characterized by the invasion of cancer cells into neighboring tissues and during the metastasis of cancer cells to distant organs [2]. In our studies of mechanisms involving adhesion-mediated signaling, we turned to Wnt-β-catenin signaling because β-catenin plays a unique dual role in the cell: on the one hand, it is a cytoskeletal plaque protein linking cadherin transmembrane adhesion receptors to the actin-cytoskeleton [3,4]; on the other hand, β-catenin is a key transducer of Wnt signaling from the cytoplasm into the nucleus as a transactivator of target genes together with the DNA binding proteins T-cell factor (TCF)/lymphoid enhancer factor (LEF) [5,6,7,8]. Activation of Wnt signaling involves the inhibition of β-catenin degradation by the ubiquitin-proteasomal system and its accumulation in the cytoplasm and in the nucleus [9,10]. The Wnt pathway has emerged during evolution as a highly conserved signaling pathway that regulates tissue morphogenesis and also tissue regeneration by its high activity in stem cells [11,12]. Aberrant activation of Wnt-β-catenin signaling (by stabilizing mutations in β-catenin, or in the adenomatous polyposis coli, APC, gene) is a hallmark of colorectal cancer (CRC) development in the majority of colon cancer patients [13,14,15]. In this review, we will describe studies on one Wnt-β-catenin target gene, the transmembrane L1 cell adhesion receptor (L1CAM, or L1) and the roles it plays in CRC progression.

## 2. L1 Family Members as Targets of Wnt/β-Catenin Signaling

**Figure 1:**
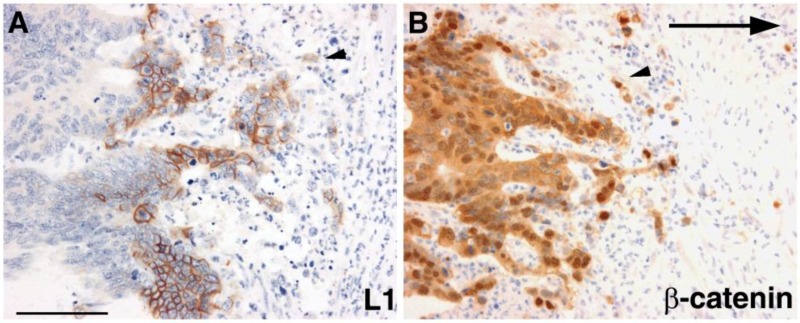
L1 is expressed in colorectal cancer (CRC) cells at the invasive edge of the tumor tissue in cells expressing nuclear β-catenin. **(A)** CRC tissue immunostaining for L1; **(B)** A serial section of the same area immunostained for β-catenin. Note the membranal staining with L1 antibody while β-catenin is stained both in the cytoplasm and in the nuclei of cells indicative of active Wnt signaling. The large arrow points to the direction of invasion. The small arrowheads point to a single invading CRC cell expressing L1 and nuclear β-catenin (from reference 30). The bar represents 75 μm.

L1 belongs to a small subfamily of the superfamily of immunoglobulin-like cell adhesion receptors that, in addition to L1, includes NrCAM, neurofascin, and NCAM [16,17,18]. These receptors were originally identified in nerve cells playing a critical role in brain development in the processes of neuronal elongation and guidance, axonal fasciculation, and path finding [19,20,21]. Point mutations along the human L1 molecule are associated with severe developmental brain diseases such as X-linked hydrocephalus, MASA syndrome, and L1 syndrome [22,23,24,25,26]. Our original efforts to identify putative β-catenin target genes in cells overexpressing a mutant (stabilized) form of β-catenin revealed, rather unexpectedly, on top of the list of genes whose level is increased, NrCAM [27]. Since NrCAM was known for its roles in neuronal cells [28], it was surprising to find its increased expression in a renal carcinoma cell line that we employed for our study. The increase in NrCAM expression in mutant β-catenin overexpressing cells was induced by the transcriptional activation of the *NrCAM* gene via TCF binding sites in the promoter of the *NrCAM* gene that were required for this activation [27]. Increased NrCAM expression was found in both human CRC tissue and melanoma that were reported to display increased β-catenin-TCF/LEF activation [27]. In a later study, where the mutant APC was reconstituted with wt APC (that normally regulates β-catenin level), L1 was among the genes whose levels were decreased [29], indicating that L1 expression depends on β-catenin signaling. We identified L1 as a β-catenin-TCF target gene in CRC cells. More significantly, we identified L1 preferentially expressed in cells at the invasive edge of human CRC tissue, together with nuclear β-catenin [30], indicative of increased β-catenin transcriptional activity (Figure 1). This finding strongly linked the elevated expression of L1 with the invasive activity of human CRC cells. Experiments manipulating L1 levels in CRC cells revealed that L1 confers an increase in cell motility and invasion and upon injection into the spleen, conferred an increase in the metastatic potential of these cells [30,31].

How can the expression of a cell adhesion receptor such as L1 be advantageous in enhancing the invasive potential of CRC cells? While L1 is categorized in contemporary cell biology textbooks as a homophilic cell-cell adhesion receptor, together with E-cadherin [32], there are large differences in the properties of these two adhesion receptors. While E-cadherin binds exclusively and very strongly to E-cadherin on the surface of adjacent cells, L1 binds rather weakly to a number of other molecules, including members of the L1 family, growth factor receptors, ECM components, and even integrins. The binding of L1 to these different partners, was shown to mediate its functions in neuronal pathfinding, fasciculation, and elongation [33]. Thus, a cell adhesion receptor that can weakly bind to a variety of extracellular ligands may constitute an ideal means for enhancing cancer cell motility. This demonstrates the essential differences in the motile properties of cells when L1 replaces E-cadherin in the same cells [34].

Since metalloproteases were also implicated in promoting the invasive potential of CRC cells by producing soluble paracrine and autocrine stimulators by cleaving the ectodomain of various receptors [35], we have analyzed ADAM10, a metalloprotease that cleaves the extracellular domain of L1 [36,37]. We have shown that ADAM10, in combination with L1, greatly enhances the invasive and metastatic potential of human CRC cells [31].

## 3. Signaling by L1 that Involves the NF-κB Pathway

**Figure 2:**
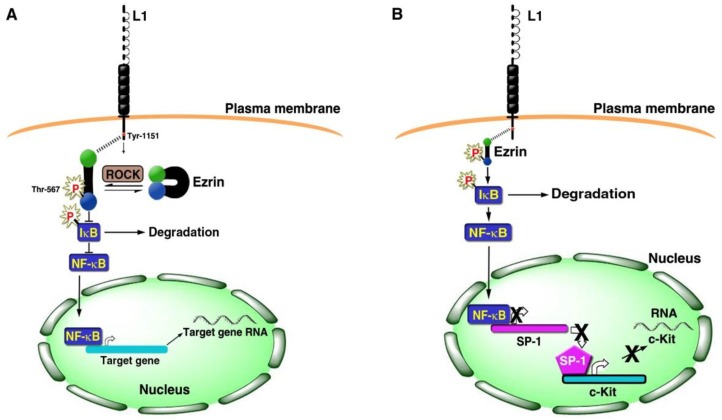
Schematic representation of L1-mediated signaling that involves ezrin and NF-κB. (**A**) Ezrin is activated by phosphorylation on threonine 567 (Thr-567) by ROCK that enables its association with the cytoplasmic tail of L1 involving tyrosine 1151 (Tyr-1151). The relocalization of ezrin in complex with L1 is involved in the recruitment of IκB to this complex, its enhanced phosphorylation and degradation by the proteasome followed by the release of NF-κB to migrate into the nucleus and activate target genes; (**B**) The L1-ezrin-NF-κB signaling is also employed in the inhibition of c-Kit transcription, indirectly, by the blocking of SP-1 transcription by NF-κB (modified from references [39,41]).

Numerous studies suggested that inflammation and CRC are related to one another [38]. We therefore examined the possibility that L1 signaling involves the NF-κB pathway and have shown that L1 confers its cancer promoting properties in CRC cells by activating the NF-κB pathway such that blocking NF-κB signaling eliminated the ability of L1 to confer its metastatic potential [39]. Activation of the NF-κB pathway requires involvement of the cytoskeletal protein ezrin that is activated by Rho-associated protein kinase (ROCK) via phosphorylation on Thr-567, followed by binding to L1 and relocalization into a submembranal complex together with L1 and IκB (Figure 2A). This complex, that includes additional components, enhances the proteasomal degradation of IκB, thereby releasing NF-κB (from its complex with IκB) and enables its nuclear localization and the activation of downstream NF-κB target genes (Figure 2A) [39].

To identify target genes that are regulated by the L1-ezrin-NF-κB pathway, we conducted gene array experiments and identified insulin-like growth factor binding protein 2 (IGFBP2) as a target of L1-ezrin-NF-κB signaling and showed that IGFBP2 transcription is directly activated by NF-κB binding to the IGFBP2 gene promoter [40]. A recent study of protein expression datasets in CRC tissue (derived from TCGA) detected high levels of IGFBP-2 in association with both disease recurrence and patient death [41]. Targeted changes in the level of IGFBP2 expression in CRC cells reproduced many of the effects conferred by changes in L1 expression in these cells, including increased motility, proliferation in the absence of serum, and enhanced tumorigenesis and liver metastasis [40]. Immunohistochemical analysis of CRC tissue samples revealed that the more invasive areas of CRC tissue exclusively included high levels of L1 and ezrin in the cell membrane, together with nuclear localization of the phosphorylated (active) subunit of NF-κB (pp65) [39]. These results strongly support a role for the L1-ezrin-NF-κB pathway in promoting CRC development.

## 4. Gene Expression Patterns of Human CRC Tissue and L1 Overexpressing CRC Cells

**Figure 3:**
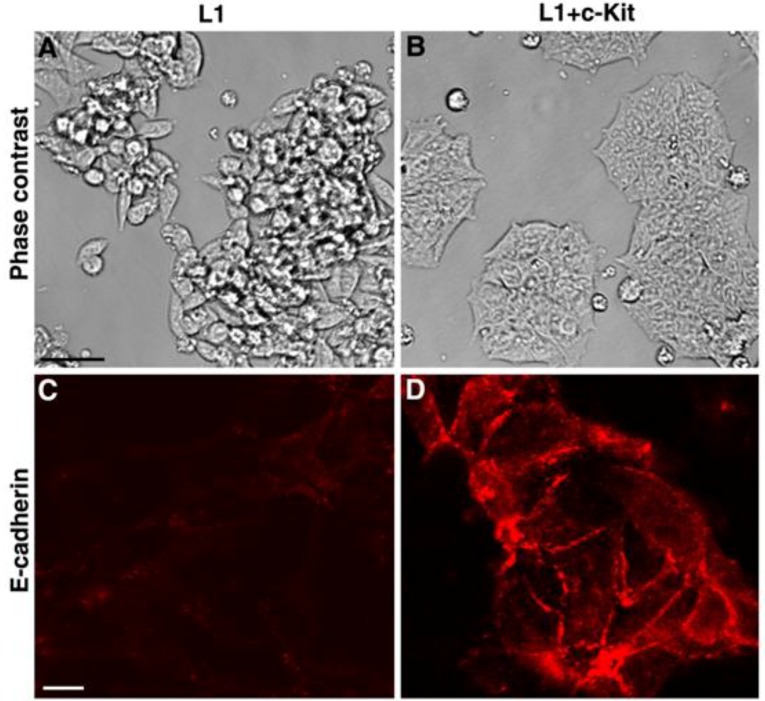
The expression of c-Kit in CRC cells induces a mesenchymal to epithelial conversion. (**A**) L1 expressing Ls174T CRC cells are organized in small three-dimensional aggregates; (**B**) The transfection of c-Kit into such cells results in cell spreading and a more epithelial-like organization of cells; (**C**) The L1 transfected CRC cells do not express significant levels of E-cadherin; (**D**) Transfection of c-Kit induces E-cadherin expression and organization at cell-cell contacts (modified from reference [42]). The bar represents 20 μm.

When comparing patterns of L1-mediated gene expression in CRC cells to gene expression patterns of human CRC tissue, both upregulated and downregulated genes were common to both [42]. When only gene expression patterns of L1-overexpressing cells that are regulated via L1-NF-κB signaling were compared to those from human CRC tissue, quite surprisingly, the c-Kit tyrosine kinase oncogenic receptor was on top of the list of genes whose levels were reduced in both L1 expressing cells and in human CRC tissue samples [42]. Since c-Kit is a well-known oncogene in a variety of tumors [43], it was rather unexpected to find that its expression is suppressed during L1-mediated CRC invasion and metastasis when cells are more aggressive and the cancer is more progressive. We found that L1 induces the binding and suppression of Sp1 by NF-κB binding and inhibition of the Sp1 promoter, a common activator of c-Kit transcription, thereby leading to a decrease in c-Kit expression [42] (Figure 2B). The overexpression of c-Kit in L1-expressing CRC cells inhibited the increase in cell motility and the liver metastasis in L1-transfected CRC cells [42]. Interestingly, in such c-Kit overexpressing cells, there was a dramatic change in cell morphology from a more aggregated and disorganized colony organization into a flat epithelial colony morphology with clear borders (Figure 3). Such cells expressed E-cadherin and displayed a decrease in a major EMT (epithelial mesenchymal transition) transcriptional regulator SLUG, reminiscent of a mesenchymal to epithelial transition (MET) [42]. In spite of the suppression of c-Kit expression in the later phases of CRC progression (invasion and metastasis), c-Kit overexpression promotes an increase in tumorigenesis in mice [42]. Such dichotomic behavior in c-Kit behavior during cancer development was also observed for another major oncogene, c-myc, during breast cancer development (promoting tumorigenesis at early phases, but being inhibitory during metastasis) [44].

## 5. L1-Mediated Gene Expression and Intestinal Stem Cell Signature Genes

**Figure 4:**
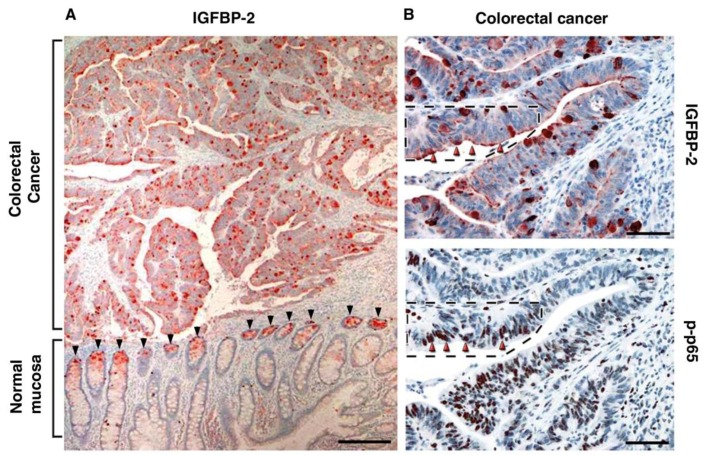
The localization of IGFBP-2 in CRC tissue. (**A**) Human CRC tissue was immunostained for IGFBP-2. The tumor tissue was strongly stained for IGFBP-2 throughout the tissue, while the adjacent normal colonic crypts were exclusively stained at the bottom of colonic crypts (black arrowheads); (**B**) Serial sections of human CRC tissue were immunostained for IGFBP-2 and the activated form of the NF-κB subunit p-p65. Note the colocalization of p-p65 in the nuclei of CRC cells together with IGFBP-2 in the membrane of the same cells (red arrowheads). The bar represents 250 μm.

**Figure 5:**
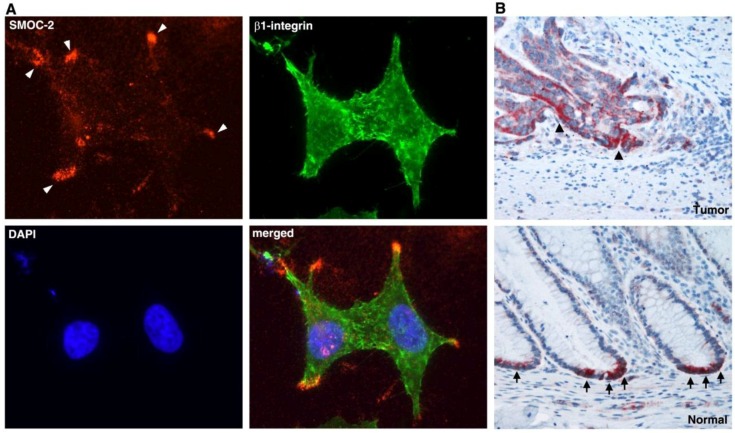
The localization of SMOC-2 (secreted, modular, matricellular, Ca^2+^-binding protein 2) in cultured CRC cells and in CRC tissue. (**A**) SMOC-2 is localized outside the cells at the tip of cellular protrusions (white arrowheads); (**B**) CRC tissue was strongly stained for SMOC-2 (black arrowheads) at the more invasive areas of the tumor, while normal colonic epithelium displayed SMOC-2 staining exclusively at the bottom of colonic crypts (black arrows), suggesting that SMOC-2 is expressed by colonic stem cells (modified from reference [51]).

While studying by immunohistochemical methods the localization of IGFBP2 in human CRC tissue samples we detected an increased expression of IGFBP2 in the tumor tissue and an exclusive localization of IGFBP2 at the bottom of colonic crypts in the adjacent normal tissue (Figure 4). The base of the intestinal and colonic crypts is the site of stem cells residence, where primarily the involvement of Wnt signaling controls intestinal homeostasis as it does in CRC development [13,45,46,47,48,49]. It was of interest to determine whether stem cell signature genes are also expressed at an increased level during L1-mediated CRC development. Comparison of a stem cell signature gene list derived from genomic and proteomic analysis in Lgr5^+^ stem cells of the mouse intestinal tissue to genes induced by L1-NF-κB in human CRC cells revealed a number of common genes [50,51]. Focusing on one of these genes, SMOC2 (secreted, modular, matricellular, Ca^2+^-binding protein 2), we found that the expression of SMOC2 is induced by an L1-ezrin-NF-κB mechanism and that SMOC2 is necessary for the L1-mediated increase in cell motility and the metastasis of CRC cells [51]. SMOC2 was secreted and organized outside the cell at the tip of cellular protrusions (Figure 5A), indicative of its involvement in cell motility [51]. More significantly, in human CRC tissue SMOC2 was preferentially localized in the more invasive areas of the tumor (Figure 5B) and exclusively at the bottom of colonic crypts of normal colonic epithelium, most probably in the stem cell compartment, as also observed in the Lgr5^+^ containing stem cells of the mouse intestine [51]. In cultured CRC cells, overexpression of SMOC2 conferred a more dispersed mesenchymal morphology and an increase in SNAIL, a major regulator of EMT that inhibits *E-cadherin* transcription. This signaling involves integrin-linked kinase (ILK) [51]. The suppression of endogenous SMOC2 in L1-expressing CRC cells reversed this process by increasing E-cadherin levels and conferring a more epithelial morphology and suppressing the metastatic potential of L1-expressing cells [51]. Together, these studies suggest that Wnt-β-catenin target genes such as L1 and their downstream effector genes can confer dramatic changes in cell morphology, motility, and metastasis by mechanisms reminiscent of EMT-like changes.

## 6. Conclusions

Wnt signaling is a key signaling pathway both in normal intestinal homeostasis and overactivation of Wnt-β-catenin signaling is characteristic of 90% of CRC patients. Here, we have reviewed our studies on one Wnt-β-catenin target gene (L1) and its functions in the progression of human CRC development. L1 was uniquely interesting because it appeared in CRC tissue, but not in the normal intestinal/colonic tissue. In addition, L1 is a cell adhesion transmembrane receptor and not a transcription factor or kinase, as are many other proteins that drive cancer. We have learned much about mechanisms by which L1 signals inside the cell, and how it interacts and affects other signaling pathways to confer changes in key cellular properties, such as cell proliferation under stress, cell motility, invasion, and metastasis. We have analyzed the relevance of our findings on L1-mediated signaling to human CRC development by analyzing human CRC tissue, by immunohistochemistry, and by performing gene array studies based on multiple tumor samples comparing them to gene expression patterns of L1-ezrin-NF-κB signaling in cultured human CRC cell lines. We discovered several key mechanisms operating during L1-mediated signaling, including the induction of intestinal stem cell signature genes. By learning about the function of such genes that are induced by adhesion-mediated signaling in CRC cells and in intestinal stem cells, we will further our understanding of normal intestinal homeostasis and also expect to provide novel targets for CRC therapy.

## Abbreviations

The following abbreviations are used in this manuscript:

L1	L1 cell adhesion molecule
APC	Adenomatous polyposis coli
IGFBP2	Insulin-like growth factor binding protein 2
SMOC2	Secreted, modular, matricellular, Ca^2+^-binding protein 2
NF-κB	Nuclear factor kappa B
IκB	Inhibitor of kappa B
EMT	Epithelial mesenchymal transition
MET	Mesenchymal epithelial transition
ILK	Integrin linked kinase
CRC	Colorectal cancer
ECM	Extracellular matrix
TCF/LEF	T cell factor/lymphoid enhancer factor
NrCAM	NgCAM-related neural cell adhesion molecule
NCAM	Neural cell adhesion molecule
ADAM10	A disintegrin and metalloprotease 10
Lgr5	Leucine-rich repeat-containing G-coupled receptor 5
Sp1	Sephacryl phosphocellulose factor 1
